# A Comprehensive Systematic Review on Functional Results, Speech and Swallowing Outcomes after Trans-Oral Robotic Surgery for Oropharyngeal Squamous Cell Cancer

**DOI:** 10.3390/jcm13206039

**Published:** 2024-10-10

**Authors:** Pierre Guarino, Francesco Chiari, Sara Cordeschi, Pasquale D’Alessio, Carla Ingelido, Giovanni Motta, Livio Presutti, Gabriele Molteni, Claudio Donadio Caporale

**Affiliations:** 1 Otolaryngology Head and Neck Unit, “Santo Spirito” Hospital, 65124 Pescara, Italy; pierreguarino@hotmail.com (P.G.); saracordeschi@live.it (S.C.); pasquale.dalessio@hotmail.it (P.D.); carlaingelido@gmail.com (C.I.); claudiodonadio.caporale@asl.pe.it (C.D.C.); 2 Otolaryngology and Audiology Unit, IRCCS Azienda Ospedaliero, Universitaria di Bologna, 40138 Bologna, Italy; livio.presutti@unibo.it (L.P.); g.molteni@unibo.it (G.M.); 3 Head and Neck Surgery Unit, University of Campania “Luigi Vanvitelli”, 80138 Naples, Italy; giovannimotta95@yahoo.it; 4 Department of Medical and Surgical Sciences (DIMEC), Alma Mater Studiorum di Bologna, 40126 Bologna, Italy

**Keywords:** OPSCC, TORS, functional outcomes, swallowing, speech

## Abstract

**Background:** Transoral robotic surgery (TORS) is nowadays considered a valuable minimally invasive approach to treat oropharyngeal squamous cell carcinoma (OPSCC). The aim of this technique is to improve functional preservation and reduce morbidity with excellent oncologic outcomes compared to the traditional transoral approach and chemoradiotherapy (CRT). The purpose of this systematic review is to assess an exhaustive overview of functional outcomes of TORS for OPSCC by evaluating several parameters reported in the available literature, such as the prevalence and dependence of tracheotomy, feeding tubes (FTs) and percutaneous endoscopic gastrostomy (PEG), the length of hospitalization, swallowing scores, speech tests and quality of life (QoL) questionnaires. **Methods**: A systematic literature review has been performed following the PRISMA 2020 checklist statement. A computer-aided search was carried out using an extensive set of queries on the Embase/PubMed, Scopus and Web of Sciences databases relating to papers published from 2007 to 2024. **Results**: A total of 28 papers were systematically reviewed, reporting 1541 patients’ data. The mean time of hospitalization was 6 days. A planned tracheotomy was performed in 8% of patients with a mean time of removal of 8 days. The prevalence and dependence of FT was 60% and 10%, respectively. Moreover, the presence of a high-stage T tumor with the contextual requirement of adjuvant therapies, the involvement of base tongues and the patient’s age being >55 years increased the risk of requiring an FT and PEG. Swallowing and long-term QoL outcomes highlight the superiority of the TORS approach alone compared to TORS with adjuvant therapies. **Conclusions**: TORS presented various favorable functional outcomes compared to other surgical approaches and primary CRT. However, adjuvant therapies after TORS strongly reduced the advantage of the robotic procedure, thus suggesting that T1 and T2 tumors may benefit mainly from TORS alone.

## 1. Introduction

Nowadays, the incidence of oropharyngeal squamous cell carcinoma (OPSCC) is steeply rising due to the increasing prevalence of oral human papillomavirus (HPV) infections [1,2]. While HPV-negative OPSCC appears to be related to smoking and alcohol, HPV-positive OPSCC usually occurs in younger patients and is characterized by a better oncological prognosis and a 5-year overall survival (OS) rate greater than 80% [3]. Conversely, the 5-year OS of HPV-negative is about 40% [4]. Over the years, OPSCC has traditionally been treated with radical surgical excision of the primary tumor using various approaches, with or without adjuvant therapy. Therefore, despite the high rates of swallowing disorders, organ-preserving therapy has been considered the gold standard of treatment for HPV-positive OPSCC by reason of higher sensitivity to radiotherapy (RT) and chemoradiotherapy (CRT) [5]. Less invasive surgical approaches, such as transoral robotic surgery (TORS) and transoral laser microsurgery (TOLM), are increasingly performed to treat OPSCC with excellent oncologic outcomes and lower morbidity than other approaches [6,7,8,9,10]. Recent literature contributions highlighted that robotic oncological outcomes for HPV-positive OPSCC are comparable to those of primary CRT [6,8]. Consequentially, the TORS approach is increasingly being used to treat HPV-positive OPSCC as a de-escalation strategy: following surgical therapy, the standard radiation dose is typically decreased to the primary site of tumors and cervical lymph nodes [7]. In terms of functional outcomes, TORS provides lower morbidity and complication rates than open surgical techniques due to a limited need for prophylactic tracheotomy, a lower rate of prevalence and dependence on a feeding tube (FT) and percutaneous endoscopic gastrostomy (PEG), faster post-operative recovery of swallowing, phonation and decreased hospital stay [11,12]. Despite the limited number of papers reporting a comprehensive analysis of functional outcomes of the robotic management of OPSCC, it is clear that swallowing and speech outcomes are highly influenced by the ability to detect the real long-term quality of life (QoL) of treated patients.

Recently, several experts in the management of swallowing have produced the European White Paper, which summarizes current evidence-based literature on dysphagia for head and neck disorders, providing recommendations to support both patients and health practitioners [13]. Moreover, the same authors also emphasize the need for robust scientific research on swallowing management, mostly for minimally surgical procedures [12]. The purpose of this systematic review is to assess an exhaustive overview of the functional outcomes of TORS for OPSCC. In particular, all the functional outcomes listed in the current literature, such as prevalence and dependence from tracheotomy, FT and PEG, hospital stays, swallowing outcomes, speech parameters and QoL questionnaires, were assessed.

## 2. Materials and Methods

### 2.1. Inclusion and Exclusion Criteria

The inclusion criteria for abstracts and full-text selection were adopted as follows: adoption of the English language, abstract and full-text availability, data on functional outcomes (prevalence and dependence from tracheotomy, FT, PEG, time of hospitalization, swallowing scores, speech tests and QoL rated in specific questionnaires) of patients who underwent TORS for OPSCC. Exclusion criteria for abstracts and full texts selection were the usage of languages other than English, the unavailability of abstracts and full texts and data of patients affected by tumors other than OPSCC.

### 2.2. Search Strategy and Information Sources

PRISMA 2020 guidelines and their checklist were applied in this systematic literature review [14]. A computer-aided search was performed using the Embase/PubMed, Scopus and Web of Science databases for articles published from 2007 to 2024. The search string for each database is reported in Table 1.

### 2.3. Study Selection and Data Extraction

**Figure 1:**
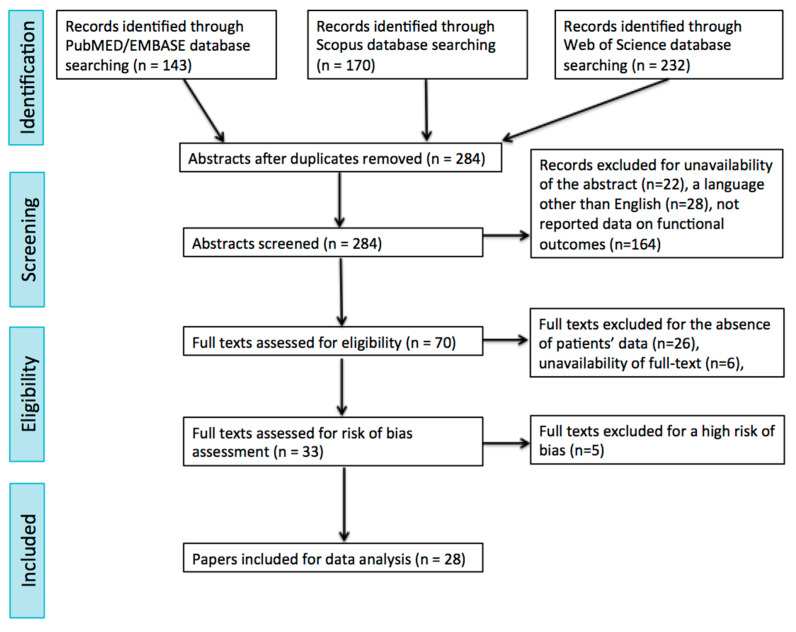
Flow chart of the study.

After running the search string listed in Table 1, the abstracts and titles obtained were screened independently by two of the authors (F.C. and P.G.), who afterward met and discussed disagreements on citation inclusion. The same two authors screened the full texts identified by such criteria, and then they met again and discussed disagreements on article inclusion. A further manual check of the references included in the articles was performed. Details on the study selection process are reported in the PRISMA flow chart (Figure 1).

### 2.4. Quality Assessment

Two authors (P.G. and F.C.), with a high level of expertise in the head and neck oncological field, independently assessed the quality of the included studies using an assessment tool for the case series and case reports [15], which considers 4 domains (selection, ascertainment, causality and reporting) and provides 8 questions to aid building up a quality score. Studies were rated as having a low, moderate or high risk of bias according to the description thereof (Table 2). The articles affected by a high risk of bias were excluded from the analysis.

### 2.5. Data Analysis

Patients’ level data were extracted and summarized. The statistical analyses were carried out through STATA v.14 (Stata Corp LLC, College Station, TX, USA). The results were expressed as the mean (standard deviation—SD) for continuous variables with normal distribution, the median (IQR) for continuous variables with non-normal distribution and percentages for categorical variables. The Shapiro–Wilk normality test was used to assess the distribution of continuous variables.

## 3. Results

Running the search strings reported in Table 1, a total of 143, 170 and 232 manuscripts were identified in the Embase/PubMed, Scopus and Web of Science databases, respectively. Subsequently, the removal of duplicates was performed, and 284 papers were thus selected. After the abstract selection, 127 articles were selected for full-text screening. The other 214 articles were excluded due to the unavailability of abstracts (22), the adoption of a language other than English (28) or a lack of reported data on functional outcomes of patients affected by OPSCC treated with TORS (164). Out of the full-text screening process, 33 papers were selected. The other 32 articles were excluded due to the absence of enough specific patient data on functional outcomes after the TORS procedure (26) or the unavailability of a full text (6). Finally, full texts were processed for a risk of bias evaluation: as a result, five articles were excluded due to a high risk of bias (Table 2). Therefore, the intended analysis could be finally carried out on 28 articles. The selection process of the papers is summarized in the PRISMA flow chart (Figure 1). The selected papers were published between 2007 and 2024. The main features of the whole group of selected articles are summarized in Table 3.

The eight questions included in the evaluation tool by Murad et al. [15] are categorized into the following four domains: selection, ascertainment, causality and reporting. Within each domain, the authors evaluated if the question-related conditions were satisfied (1) or not (0). Final results differentiated case reports or series into three categories of risk of bias as follows: low risk (L) if all questions of the four domains were matched; intermediate risk (I) if questions of three out of the four domains were matched; high risk (H) if questions of less than three out of the four domains were matched.

### 3.1. Population Characteristics

A total of 1541 patients’ data reported in 28 different papers were systematically reviewed (Table 3). The largest and smallest study population was made up of 267 and 9 patients, respectively. There were 87% males and 13% females, with a mean age of 57.5 years. The most involved site of OPSCC is the tonsil (54%), followed by the base tongue (45%) and the soft palate (1%). Data on the pathological tumor stage were stratified as follows: early stage, which included T1 and T2 tumors (82%), according to the TNM of oropharyngeal tumors eighth edition of the American Joint Committee (AJCC) [45], and advanced stage, which included T3 and T4 tumors (18%). There were four different studies that exclusively included patients affected by early-stage tumors; one single study analyzed the functional outcomes of advanced-stage OPSCC. A pathological lymph node involvement was described in 60% of patients. Information about a relationship with HPV was reported in 18 papers.

**Table 3:** Patients’ features and functional outcomes on prevalence and dependence of tracheotomy, feeding tube and PEG, oral intake and surgical complications. The same table analyzed tools, objectives and outcomes and results of swallowing, speech and quality of life outcomes.

Authors (y of Publication)	Nr of pts Males % Mean Age	Site (% pts)	Stage (% pts)	HPV pos p16 pos % pts	Treatment (% pts)	Mean FU Time	Mean HS	Tracheo % of pts, Rate and Mean Time of Removal	FT % of pts, Rate and Mean Time of Removal	PEG % pts, Rate and Mean Time of Removal	Oral Intake after Surgery % pts	Surgical Complications	Tools	Objectives	Outcomes and Results
Achim et al. (2017) [16]	74 92% 61 y	BPOT (42%) TO (57%) SP (1%)	Early T (99%) Adv T (1%) N pos (86%)	NA 95%	TORS alone (27%) TORS + RT (42%) TORS + CRT (31%)	21 m	4.8 d	1% NA 6 w	100% 100% 9.8 d	NA NA NA	NA	NA	EAT-10, HNQOL and PSS-HN	Long-term functional outcomes after TORS	HNQOL eating subscale demonstrated significant short-term worsening post-op with a more rapid and complete recovery in pts treated with TORS alone. At long FU pts treated with TORS alone had EAT-10 scores not significantly different from baseline despite scores remaining significantly below baseline on HNQOL eating subscale. HNQOL speech and pain domains highlighted a significant decrease in scores post-op followed by a recovery no longer different from baseline. HNQOL emotional subscale was relatively stable over time.
Charters et al. (2021) [19]	25 88% 64 y	BPOT (44%) TO (56%)	Early T (96%) Adv T (4%) N pos (80%)	88% NA	TORS alone (24%) TORS + RT (64%) TORS + CRT (12%)	NA	NA	NA NA NA	NA NA 76.2 d	NA NA NA	NA	NA	FEES, EAT-10, MDADI, PSSHN and SHI	Swallowing outcomes following TORS alone, TORS + RT/CRT and primary RT	FESS at 12 m post-treatment revealed best outcomes for pts treated with TORS respect to RT: ability to achieve sustained vocal fold closure (76% vs. 45%), secretion management (60% vs. 25%), laryngeal penetration (40% vs. 45%) and laryngeal aspiration (16% vs. 18%). No differences were observed on quality of life scores. Mean SHI was similar between TORS and RT (13.8 vs. 12.9 and 14.1 vs. 17.1) in matched and unmatched analysis of functional outcomes, respectively.
Dean et al. (2015) [20]	22 86% 62 y	BOT (41%) TO (52%) SP (7%)	Early T (45%) Adv T (55%) N pos (50%)	NA NA	NA	NA	3 d	4% 100% 4 d	NA NA NA	NA NA NA	NA	Bleeding 8% Neck abscess 4%	NA	NA	NA
Dziegielewski et al. (2013) [21]	81 80% 31 y	BPOT (20%) TO (80%)	Early T (90%) Adv T (10%) N pos (89%)	72% NA	TORS alone (13%) TORS + RT (25%) TORS + CRT (62%)	22.7 m	NA	1% 100% NA	21% 71% NA	NA NA NA	100% at discharged time	OPC fistula 2%	HNCI	Long-term functional outcomes of TORS for OPSCC	All HNCI scores declined at 3 m post TORS. However, eating function was the most affected domain at 12 m after TORS with a large diminution in a statistically significant way. Speech and aesthetic function had a small diminution to baseline. Attitude was the most affected HNCI domains at 12 m after TORS with a large diminution in a statistically significant way.
Hughes et al. (2023) [23]	116 NA 57 y	BPOT (22%) TO (75%) SP (3%)	Early T (97%) Adv T (3%) N pos (86%)	100% 100%	TORS alone (18%) TORS + RT (54%) TORS + CRT (28%)	40.6 m	NA	4% NA NA	NA NA NA	NA NA NA	NA	NA	FOIS, weight loss, FT prevalence and FT dependence	Swallowing function after primary RT vs. TORS for OPSCC HPV related	Mean FOIS scores in the TORS group were 6.9 at baseline and 6.4 at 1 y, compared with 6.7 and 5.6 for RT group. Only clinical nodal stage was found to be associated with FOIS change. FT prevalence and dependence rates were higher in RT group.
Hutchenson et al. (2019) [25]	75 87% 60 y	BPOT (45%) TO (51%) SP (4%)	NA	NA NA	TORS alone (31%) TORS + RT (49%) TORS + CRT (20′%)	NA	NA	NA NA NA	NA NA NA	NA NA NA	NA	NA	MDADI, MBS and DIGEST	Comparison on dysphagia measures between primary TORS and nonsurgical treatment for OPSCC with low and intermediate risk	Only 22.7% pts developed moderate-severe pharyngeal dysphagia (DIGEST >2) in the acute post-surgical period that improved, but did not recover to baseline by 3–6 months. DIGEST improved by 3 to 6 m but remained worse than at baseline: at 3 m to 6 m pts with DIGEST > 2 were 7% treated with TORS and 16% treated with RT. Post-treatment dysphagia grades according to DIGEST did not significantly differ between pts treated with primary TORS and RT. At the start of RT MDADI swallowing symptom severity score were significantly worse in the post-TORS group compared with post-induction change and treatment naïve pts.
Ji et al. (2024) [6]	41 90% 58 y	BPOT (32%) TO (67%) SP (1%)	Early T (97%) Adv T (3%) N pos (71%)	NA 73%	TORS alone (15%) TORS + RT (44%) TORS + CRT (41%)	NA	NA	15% 100% NA	29% NA NA	NA 98% NA	NA	Neck seroma 15% Hematoma 3%	MBS, DIGEST and KSMST	Long-term functional swallowing outcome after TORS	MBS was measured through DIGEST score as 0 (25%), 1 (69%), 2 (7%), 3 (0%) and 4 (0%). Only 7% pts showed an abnormal value on mean tongue motility score (15.5), 0% in other scores, as mean articulation score (5.9), mean verbal diadochokinesis (25.1) and reading speed (11.9).
Genden et al. (2011) [22]	31 80% 61 y	BPOT (45%) TO (55%)	Early T (87%) Adv T (13%) N pos (84%)	NA NA	TORS alone (80%) TORS + RT (10%) TORS + CRT (10%)	18 m	NA	0% NA NA	19% 100% 6 d	22% 100% NA	NA	OPC fistula 7%	PSS and FOIS	Role of reconstruction for TORS pharyngectomy and concomitant neck dissection	PSS demonstrated progressive improvement in mean scores at 2 w, 2 m, 6 m, 9 m and 1 y for eating in public and diet. Same results were observed for FOIS score. Of these 31 cases, a muscle-mucosal flap and free flap were used in 25 and 9 pts, respectively.
Lee (2014) [27]	27 78% 58 y	TO (100%)	Early T (82%) Adv T (18%)	67% NA	NA	20.3 m	14.6 d	59% 100% 5 d	NA NA 9 d	0% NA NA	NA	None	MDADI	Comparison of functional outcomes among TORS vs. conventional open surgery	MDADI results measured at 12 m post-op revealed that TORS group had a significantly better subjective swallowing status than open surgery group.
Leonhardt et al. (2010) [28]	38 73% 57 y	NA	Early T (89%) Adv T (11%) N pos (74%)	NA NA	TORS alone (25%) TORS + RT (57%) TORS + CRT (18%)	15.2 m	NA	3% NA NA	NA NA NA	39% 98% NA	NA	NA	PSS and SF-8	Functional results of patients affected by OPSCC treated with TORS	PSS eating and diet domains significant declined (*p* < 0.001) at 6 m after treatment. SF-8 bodily pain and global health domains significant decrease at 6 m (*p* = 0.019 e *p* = 0.049) and returned to baseline at 1 y (*p* = 0.947 and *p* = 0.968). A post-operative RT appears to have a greater impact on swallow function at 6 m, which returned normal at 1 y. The addition of CHT had a greater impact on swallowing at 6 m. PSS speech domain significant declines (*p* < 0.001) at 6 and 12 m. Pts treated with TORS alone had a little impact of resection on speech.
Li et al. (2023) [30]	83 85% 57 y	BPOT (25%) TO (64%) SP (11%)	Early T (100%) N pos (61%)	30% NA	TORS alone (72%) TORS + RT (6%) TORS + CRT (22%)	29.5 m	5 d	14% 100% 9 d	100% NA 14.5 d	NA NA NA	NA	Bleeding 2% Dehiscence 1%	NA	NA	NA
Lorincz et al. (2014) [29]	35 74% 65 y	BPOT (40%) TO (51%) SP (9%)	Early T (97%) Adv T (3%) N pos (74%)	34% 51%	TORS alone (37%) TORS + RT (40%) TORS + CRT (23%)	13 m	NA	14% 100% 11 d	100% 100% 5 d	46% 100% 29 d	NA	Bleeding 6%	NA	NA	NA
Moore et al. (2009) [32]	45 89% 57 y	BPOT (58%) TO (42%)	Early T (71%) Adv T (29%) N pos (84%)	NA NA	TORS alone (26%) TORS + RT (56%) TORS + CRT (18%)	12.3 m	3.8 d	31% 100% 7 d	49% 100% 12.5 d	18% NA 140 d	50% at 1 d 89% at 4 d	OPC fistula 7% Hematoma 3%	FOSS and CS	Functional assessment of swallowing in pts underwent to TORS for OPSCC	All pts who had FOSS score >2 after completing therapy had BOT tumors and underwent post-operative CRT. All pts underwent to TORS alone presented FOSS score of 0 without 4 w after surgery. Speech was normal with communication of 0 in all 45 pts post-op at first evaluation. 8% pts had rhinolalia when they were dismissed from hospital, but it resolved rapidly.
Moore et al. (2012) [33]	66 89% 55 y	NA	Early T (85%) Adv T (15%) N pos (87%)	72% 89%	TORS alone (16%) TORS + RT (21%) TORS + CRT (62%)	36 m	NA	26% 98% NA	47% 97% NA	27% 95% 140 d	NA	OPC fistula 6% Bleeding 1%	NA	NA	NA
Nichols et al. (2019) [34]	34 82% 58 y	BPOT (29%) TO (71%)	Early T (88%) Adv T (12%) N pos (71%)	NA 88%	TORS alone (29%) TORS + RT (47%) TORS + CRT (24%)	29 m	NA	NA NA NA	NA NA NA	NA NA NA	NA	NA	MDADI	RT vs. TORS for OPSCC	MDADI total scores at 1 y were mean 86.9 in RT group and 80.1 in TORS group. Pts receiving total oral diet with no restriction at 1 y were 100% in RT group and 84% in TORS group. Pts treated with RT presented superior swallowing-related scores 1 y after treatment, although the difference did not represent a clinically meaningful change.
Olsen et al. (2013) [35]	20 67% 61 y	BPOT (33%) TO (67%)	Early T (94%) Adv T (6%) N pos (28%)	56% 72%	TORS alone (100%)	NA	3.1 d	17% 100% 10 d	44% NA 13.6 d	0% NA NA	100% at 4 w	None	FOSS	Functional results of TORS for OPSCC HPV related	At last FU 78% pts had normal swallowing function (FOSS 0) and 22% had mild dysphagia (FOSS 1).
Salmon et al. (2021) [36]	9 78% 64 y	BPOT (100%)	Early T (100%) N pos (78%)	55% NA	TORS alone (42%) TORS + RT (36%) TORS + CRT (22%)	NA	NA	0% NA NA	NA NA NA	NA NA NA	NA	NA	FOIS, MILP and EAT-10	Functional swallow-related outcomes following TORS for base of tongue carcinoma.	A significant difference was seen between pre-op FOIS score and 1 w after surgery (*p* = 0.016). MILP values significantly declined from pre-op to 1 w after surgery (*p* = 0.0001) with a complete recovery after 1–3 m (*p* = 0005). Significant differences in EAT-10 scores were observed between pre-op and 1 w after surgery (*p* = 0.0001), 1 w and 4 w after surgery (*p* = 0.001) and 1 w and 12 w after surgery (*p* = 0.0002).
Scott et al. (2023) [37]	31 71% 59 y	BPOT (19%) TO (71%) SP (10%)	Early T (100%) N pos (48%)	77% 77%	TORS alone (68%) TORS + RT (16%) TORS + CRT (16%)	NA	NA	NA NA NA	NA NA NA	NA NA NA	NA	NA	FEES, MDADI, EORTC-C30 and HeN35	Comparison on functional outcomes in pts treated with primary TORS and primary RT	Objective improvements were observed on FEES and MDADI score from 1 y to 3 y FU. With regards to DIGEST improvements were seen in efficiency scores (pharyngeal residue and pattern of residue). There was no clinically significant change in any subscale or symptom scale for pts treated with TORS. However, here was only a clinically meaningful in the sticky saliva
Sethia et al. (2017) [38]	111 83% 57 y	BPOT (17%) TO (71%) SP (12%)	Early T (94%) Adv T (6%) N pos (86%)	68% 78%	TORS alone (12%) TORS + RT (27%) TORS + CRT (61%)	NA	NA	0% NA NA	NA NA NA	44% NA 130 d	NA	NA	HNCI	Swallowing outcomes of TORS with or without adjuvant therapy for OPSCC	HNCI eating domains highlighted how pts underwent to CRT had significantly lower scores at baseline (*p* < 0.01) and 3 m after treatment (*p* = 0.01). Eating domains were significantly higher than for adjuvant RT at 3 m (*p* < 0.01). HNCI speech domains for TORS alone were significantly higher than for adjuvant CRT at 3 m (*p* = 0.04) and for adjuvant RRT at 6 m (*p* = 0.03) post-surgery.
Sharma et al. (2016) [39]	58 95% 58 y	BPOT (59%) TO (41%)	Early T (87%) Adv T (13%) N pos (87%)	77% NA	TORS alone (11%) TORS + RT (61%) TORS + CRT (28%)	24 m	NA	NA NA NA	33% NA NA	NA NA NA	NA	NA	NA	NA	
Shenouda et al. (2019) [40]	21 66% 66 y	BPOT (29%) TO (71%)	Early T (100%) N pos (62%)	58% NA	TORS alone (28%) TORS + RT (48%) TORS + CRT (24%)	NA	7 d	10% 100% 5 d	38% 100% 10 d	0% NA NA	NA	Bleeding 12% OPC fistula 4% Flap necrosis 4%	NA	NA	NA
Sievert et al. (2020) [41]	24 71% 61 y	BPOT (50%) TO (50%)	Early T (91%) Adv T (9%) N pos (50%)	NA NA	TORS alone (25%) TORS + RT (29%) TORS + CRT (46%)	37.9 m	16.6 d	41% 96% 5 d	NA 92% NA	54% NA 200 d	NA	Bleeding 12%	NA	NA	NA
Sinclair et al. (2011) [16]	42 69% NA	BPOT (31%) TO (69%)	Early T (100%) N pos (76%)	NA NA	TORS alone (24%) TORS + RT (45%) TORS + CRT (31%)	17 m	1 d	0% NA NA	2% 100% NA	18% 100% NA	NA	NA	MDADI	Patient perceived and objective functional outcomes following TORS	40% pts improved or unchanged pre-op MDADI score during immediate visit and 26% pts had an identical global immediate MDADI post-op and at last FU. N status (*p* = 0.049), FU < 12 m (*p* = 0.03), pre-op physical score < 100 (*p* = 0.01) predicted poor physical outcomes; pos surgical margins (*p* = 0.03) predicted poor functional outcomes. Poorer pre-op MDADI scored not predict FT retention.
Van Abel et al. (2019) [10]	267 89% 58 y	BPOT (34%) T (66%)	Early T (88%) Adv T (12%) N pos (58%)	100% 100%	TORS alone (25%) TORS + RT (30%) TORS + CRT (45%)	NA	NA	12% 99% 5 d	NA NA NA	30% 99% 120 d	NA	NA	PSS-HN, MBS, FOIS	Comparison of outcomes between pts with OPSCC HPV related treated with TORS alone, TORS + RT and TORS + CRT	The percentage of pts reporting a total oral diet with no restrictions on FOIS dropped from 20% to 5% following RT and from 26% to 12% following CRT. Aspiration for liquid at any point during swallowing attempt on MBS was similar before and after RT, but increased after CRT (19% vs. 28%). PSS-HN dropping from 55% to 13% following RT and from 45% to 19% following CRT. There was a substantial impact on swallowing outcomes following standard adjuvant therapies. Speech measures were very good overall but did demonstrate worsening function following standard adjuvant therapies. Hoarseness nearly doubled for both TORS + RT (32% to 56%) and TORS + CRT (24% to 45%).
Weinstein et al. (2007) [42]	27 93% 56.7 y	BPOT (49%) T (49%) SP (2%)	Early T (78%) Adv T (22%) N pos (85%)	NA NA	TORS alone (11%) TORS + RT (33%) TORS + CRT (56%)	18 m	NA	8% 100% 2.7 d	100% 96% NA	63% NA NA	NA	Trismus 7% Bleeding 4%	NA	NA	NA
Weinstein et al. (2010) [9]	47 91% 56.7 y	BPOT (49%) T (49%) SP (2%)	Adv T (100%) N pos (98%)	NA NA	TORS alone (5%) TORS + RT (31%) TORS + CRT (64%)	26 m	NA	10% 100% NA	NA NA NA	NA NA NA	NA	None	NA	NA	NA
Weinstein et al. (2012) [43]	30 70% 59 y	BPOT (30%) T (50%) SP (20%)	Early T (83%) Adv T (17%) N pos (50%)	NA NA	TORS alone (100%)	33 m	3.6 d	3% NA NA	NA NA NA	NA NA NA	NA	Bleeding 6% Joint capsulitis 3%	NA	NA	NA
White et al. (2016) [44]	61 79% 61 y	NA	Early T (92%) Adv T (8%) N pos (51%)	NA NA	TORS alone (100%)	NA	3.8 d	23% NA NA	35% NA NA	NA NA NA	NA	Infections 10%	NA	NA	NA

Adv T = pT3 and pT4 stage according to VIII ed AJCC; BPOT = base of tongue; CS = communication score; CRT = chemoradiotherapy; d = day; DIGEST = dynamic imaging grade of swallowing toxicity; Early T = pT1 and pT2 stage according to VIII ed AJCC; EAT-10 = eating assessment tool; FEES = fiberoptic endoscopic evaluation of swallowing; FOIS = functional oral intake scale; FOSS = functional outcomes swallowing score; FU = follow up; HNCI = head and neck cancer inventory; HNQOL = head and neck quality of life; OPC = oropahryngocutaneous; KSMST = Korean Speech Mechanism Screening test; m = month; MDADI = M.D. Anderson Dysphagia Inventory composite score; MILP = maximum isometric lingual pressure; MBS = modified barium swallow; NA = not available; OPSCC = oropharyngeal squamous cell carcinoma; PEG = percutaneous endoscopic gastrostomy; pos = positive; PSS-HN = performance status scale head and neck; PSSHN = public status scale head and neck Pts = patients; RT = radiotherapy; SHI = speech handicap index; SF-8 = short form health survey; SP = soft palate; TO = tonsil; Tracheo = tracheotomy; y = year; w = week.

### 3.2. Therapeutic Management, Surgical Complications, Time of Hospitalization and Follow-Up

Patients’ data on therapeutic management, the mean time of hospitalization and the mean follow-up time are also summarized in Table 3. The whole group of patients (100%) underwent a TORS procedure with or without adjuvant therapy based on RT or CRT. In particular, the “TORS alone” procedure was described in 28% of cases, while “TORS with adjuvant RT” was reported in 48% of cases and “TORS with adjuvant CRT” in 24% of patients. The most frequent surgical complication was post-operative bleeding (range 0–12%), followed by oro-pharyngeal-cutaneous fistula (0–7%) and neck abscess (0–6%). The mean hospitalization time was 6 days (range 1–16 days), and the mean follow-up time was 25 months (range 12.3–40.6 months).

### 3.3. Tracheotomy, Feeding Tube, Percutaneous Endoscopic Gastrostomy and Oral Intake

Twenty-three studies disclosed information on the prevalence and dependence of tracheotomy and the relative mean time of removal (Table 3). The average rate of removal of the tracheostomy tube was 99% (range 96–100%). Temporary tracheotomy was performed in 8% of patients (range 0–59% per paper). Moreover, the mean time of removal of tracheotomy was 8 days (range 1 day–6 weeks per paper).

Seventeen studies described findings on the prevalence, dependence and mean time of removal of FT (Table 3). FT was applied in 60% of patients (range 2–100% per paper) with a mean removal rate of 90% (range 72–100% per paper) and a consequential 10% dependence rate. The dependence rate from FT was mostly related to the need for adjuvant therapy (95% of patients with FT at last FU). However, patients with temporary FT presented a mean time of removal of 18 days (range 6–72.6 days per paper).

Fourteen studies reported information on the prevalence, dependence and mean time of removal of PEG (Table 3). PEG application was performed, on average, in 30% of patients (range 22–63% per paper), with a mean removal rate of 98% (range 95–100% per paper) and a mean removal time of 120 days (range 29–200 days per paper). At the same time, the dependence rate from PEG was exclusively related to adjuvant therapies, as just reported on FT dependence.

In conclusion, three studies reported some insights on the oral intake. Two different papers reported how their own cohorts of patients were able to eat an oral diet at their hospital discharge. Another study stratified the oral intake of patients, describing 50% and 89% of cases with a full oral intake one day and four days after surgical procedures, respectively.

### 3.4. Swallowing Parameters, Speech Outcomes and Quality of Life Questionnaires

In Table 3, data are reported on 17 papers analyzing swallowing parameters and relative dysphagia scores. Many different scores and tools were utilized to examine patient’s swallowing after TORS, such as the dynamic imaging grade of swallowing toxicity (DIGEST), eating assessment tool (EAT-10), EORTC-C30, fiberoptic endoscopic evaluation of swallowing (FESS), functional oral intake scale (FOIS), functional outcomes swallowing score (FOSS), FT prevalence and dependence, head and neck cancer inventory (HNCI), head and neck quality of life—eating subscale (HNQOL)—modified barium swallowing (MBS), M.D. Anderson Dysphagia Inventory composite score (MDADI), maximum isometric lingual pressure (MILP), penetration aspiration scale (PAS) and performance status scale for head and neck patients (PSS-HN).

Conversely, Table 3 summarizes information on speech outcomes and QoL questionnaires. In particular, speech functions after TORS were analyzed by eight papers through different scores and tools as follows: communication score (CS), head and neck cancer inventory (HNCI), head and neck quality of life—speech subscale (HNQOL)—Korean speech mechanism screening test (KSMST) and speech handicap index (SHI).

In conclusion, data from five different papers are displayed in Table 3, which analyzed short-term and long-term questionnaires dealing with QoL, such as head and neck cancer inventory (HNCI), head and neck quality of life (HNQOL), public status scale head and neck (PSSHN) and short-form health survey (SF-8).

## 4. Discussion

HPV-related cancers now account for roughly 70% of new OPSCC cases, with an excellent prognosis, resulting in a population of cancer survivors with great longevity and a propensity for late treatment-related sequelae [23]. Traditionally, concurrent CRT was recognized as the most effective treatment for HPV-positive OPSCC [39]. However, nonsurgical treatments are characterized by several side effects. Sharma et al. [39] described that 43% of patients with HPV-positive OPSCC were treated with CRT, resulting in severe toxic effects, the most common of which was pharyngeal dysfunction. Moreover, cooperative group trials and population-based data suggested an excess burden of dysphagia in HPV-positive OPSCC treated with CRT compared to other modalities [25]. Indeed, some studies highlighted how potential long-term radiation damage had a deep impact on social functioning and mental health [46]. Patients with HPV-negative OPSCC usually presented worse outcomes than their counterparts. Treatment resistance of HPV-negative OPSCC has raised the question of whether a more aggressive approach incorporating primary surgery followed by RT, with or without CRT, might lead to better outcomes than those associated with CRT alone [47]. Jackson et al. [48] underlined how patients with HPV-negative OPSCC treated by TORS presented favorable oncological outcomes with better functional results than those reported during other surgical approaches, especially in the early stages of the disease. Indeed, Lechner et al. [49] reviewed the main approaches for OPSCC, emphasizing that TORS is strongly recommended to equal oncological outcomes for early-stage OPSCC tumors compared to open surgical approaches (e.g., mandibulectomy and transcervical pharyngectomy) or nonsurgical approaches (i.e., RT and CRT). Although the introduction of TORS has allowed surgical resection of OPSCC without the functional side effects of open approaches, a limited group of patients with an advanced stage of OPSCC should not be candidates for TORS procedure [39]. Therefore, Weinstein et al. indicated eligible TORS-only patients with early-stage OPSCC tumors as T1, T2 and selected T3 [43]. According to the last-mentioned results, 82% of cases of our cohort of patients presented an early-stage tumor. The increasing awareness of functional outcomes after TORS treatment was markedly notable, and several studies detected patients’ changes in their QoL associated with the disease. The side effects of treatment included difficulty in speech, breathing and eating, as well as the psychological impact of the loss of function and physical disfigurement [28].

Prevalence and dependence from tracheotomy, FT and PEG were the most commonly reported functional outcomes after TORS [1]. Several authors employed such parameters to compare functional outcomes between TORS and conventional surgical techniques, such as a transoral approach or mandibulectomy, showing better functional outcomes for robotic management [20,27,44]. In particular, Lee et al. [27] reported that patients treated with TORS for OPSCC T1-T3 and those undergoing open surgery for the same T-stage tumors demonstrated a full swallowing ability after a mean of 6.5 days and 16.7 days, respectively. Furthermore, the mean time of removal of the tracheotomy was significantly in favor of robotic approaches (5 days vs. 13.2 days) [27]. A reasonable explanation for that is related to the injury to the mouth floor muscles, constrictor muscles and pharyngeal nerve plexus, which are minimized during TORS procedures. Furthermore, Dean et al. [20] and White et al. [44] reported a reduction in the mean length of hospital stay in patients undergoing TORS (5 days and 3.8 days) compared to open resection (8.2 days and 8 days). The systematic review analysis evidenced that temporary tracheotomy was performed in 8% of patients, with a mean rate of removal of 99% and a mean time of removal of 8 days. The rate of temporary tracheotomy varies according to surgical preferences, with a range of 0–59% per paper. Subsequently, Lee et al. [27] reported a higher rate of planned tracheotomies (59% patients). Nevertheless, the same authors [27] announced that 100% of patients removed the tracheotomy without complications, with a low rate of mean time of removal (6 days). The tumoral stage, extension and localization influenced the physician’s choice of performing a temporary tracheotomy. Accordingly, a planned tracheotomy was more likely performed during TORS in association with bilateral neck dissection, the base of tongue tumors and higher T stage [9]. To date, tracheotomy dependency is considered a very rare entity: only two patients [33,41] presented a permanent tracheotomy due to adjuvant therapy complications as persistent pharyngeal edema with consequent respiratory failures. Moreover, Yeh et al. [50] compared the tracheotomy dependency rate between primary RT and TORS for OPSCC, reporting non-significant differences (0–4.5% for RT and 0–3.5% for TORS).

Regarding FT parameters, this systematic analysis highlighted that FT was applied in 60% of patients, with a mean rate of removal of 90% and a mean time of removal of 18 days. The application of FT depended on several patient parameters and surgical behaviors, resulting in a high range of FT placement (2–100%). Thereby, some authors [29,51] preferred to place FT in all patients undergoing TORS, while other authors chose to select cases that needed FT [16].

The full oral intake after surgical TORS was considered a challenging topic for surgeons. Dziegielewski et al. [21] reported that the whole group of patients of their cohort treated with TORS alone was discharged home with a full oral diet, without FT. Moore et al. [32] reported that 50% of their patients started an oral intake one day after TORS in 50% of patients and 89% of cases in the first four days. Conversely, other surgeons quoted a return to oral intake swallowing times of approximately 20–40 days [35]. The very high heterogeneity for starting an oral diet intake and FT prevalence revealed the lack of guidelines and an overall consensus about the management of swallowing in such patients. However, it is known that objective swallowing ability deteriorated during adjuvant therapy due to the longer duration of treatment with associated long-term side effects, as acute mucositis, odynophagia and dysphagia severely limited oral intake [19]. Dziegilewski et al. [21] found that older patients (age > 55 years) were nearly five times more likely to require FT after TORS than younger patients. Furthermore, if the TORS resection included >1 oropharyngeal subsite, patients were 5.6 times more likely to require FT, and patients with pT3 or pT4 tumors were 27 times more likely to not be weaned from FT. This underscores how patient age and tumor extent were related to FT prevalence and dependence. Similarly, post-operative CRT was found to be associated with an increased risk of FT dependency for more than 3 months [51].

The current systematic analysis revealed that PEG was performed in 30% of patients, with a mean removal rate of 98% after an average of 120 days. Moore et al. [33] suggested that patients requiring PEG were closely associated with an advanced stage of the tumor, the involvement of the base tongue and post-operative CRT. For this reason, Weinstein et al. [9] cautioned against resecting more than 50% of the base tongue to prevent dysphagia, suggesting the use of PEG in the case of high resection of the base tongue. Langmore et al. [52] retrospectively evaluated the swallowing function of patients who underwent adjuvant RT after TORS who did and did not receive a prophylactic PEG, revealing that the higher incidence of PEG in the RT cohort may, therefore, be attributed to swallowing disuse and dysphagia.

Studies comparing the functional outcomes of TORS and RT/CRT modalities for OPSCC used MDADI scores with contrasting results. More et al. [31] found superior MDADI scores in the RT group; however, the phase-two randomized ORATOR trial [34] found superior MDADI scores in the RT group without being clinically meaningful. In particular, patients receiving a total oral diet with no restriction at 1 year were 100% in the RT group and 84% in the TORS group, and the total mean MDADI scores at 1 year were 86.9 in the RT group and 80.1 in the TORS cohort. Sinclair et al. [16] described that the N status (*p* = 0.049), follow-up <12 m (*p* = 0.03), pre-operative MDADI score < 100 (*p* = 0.01) and positive surgical margins (*p* = 0.03) predicted poor physical outcomes. Hutcheson et al. [25] completed a retrospective secondary analysis of dysphagia following TORS or RT using videofluoroscopy and a DIGEST rating, revealing a high number of patients with moderate-to-severe dysphagia in the RT/CRT group compared to the TORS one, without significant differences. Charter et al. [19] analyzed 49 patients through FESS 12 months after treatment, highlighting better outcomes for patients treated with TORS compared to RT. Moreover, Lee et al. [27] compared functional outcomes between TORS and conventional open surgery through MDADI scores measured 12 months after surgical procedures, evidencing that the TORS group had a significantly better subjective swallowing status than the open surgery one. Of note, adjuvant therapies strongly influenced swallowing outcomes. Moore et al. [32] found that all patients who had a FOSS score > 2 after completing their therapy were affected by base tongue tumors and treated with adjuvant CRT. Conversely, patients who underwent TORS alone presented a FOSS score equal to 0 within 4 weeks after TORS. Sethia et al. [38] revealed that patients having undergone CRT after TORS had significantly lower scores of HNCI—eating domains at the baseline (*p* < 0.01) and 3 months after treatment (*p* = 0.01). Furthermore, Leonhardt et al. [28] demonstrated that for patients treated with TORS alone, the impact of resection on the swallowing function was minimal compared to the baseline, and the addition of post-operative adjuvant RT or CRT appeared to have a greater impact on the swallowing function at 6 months, which returned to near baseline levels by 12 months. Consequently, the current literature reported a clear superiority of TORS compared to open surgical procedures and nonsurgical treatments in terms of functional results. However, the need for adjuvant therapies seems to worsen every swallowing outcome with a difficult recovery to the baseline.

Several studies analyzed the speech function of patients undergoing robotic procedures for OPSCC through different tests and scores and disclosed that there was only a small and non-significant decline with respect to the baseline [6,10,19,21,28,33,38,51]. Moore et al. [32] reported that 8% of patients had rhinolalia at their dismissal from the hospital, but it was resolved rapidly. Van Abel et al. [10] demonstrated a non-statistically significant worsening function following standard adjuvant therapies.

A high heterogeneity of QoL questionnaires was utilized to analyze long-term functional outcomes. A prospective study by Achim et al. [16] describing long-term QoL outcomes revealed that patients undergoing TORS alone had better QoL parameters, which returned to near the baseline, compared to patients undergoing TORS with adjuvant therapies. Sethia et al. [38] and Scott et al. [37] studies led to similar conclusions, evidencing the negative effect of RT and CRT on long-term patients’ QoL.

This is the most recent and exhaustive systematic review to overview functional outcomes of TORS for OPSCC, evaluating all parameters reported in the current literature. This analysis included several studies reporting a heterogeneous set of scores and different tools to assess the swallowing function, phonation and QoL, which did not allow for statistical analysis. However, the authors reported a comparison between TORS and the more conventional surgical approach in order to support the newly known evidence of the superiority of the first technique over the others in terms of functional results [53]. However, the authors made a larger effort to compare the same parameters between TORS and primary CRT due to the lack of significant evidence in terms of functional outcomes. These results firstly demonstrate that the robotic procedure presents several advantages in swallowing and long-term QoL compared to CRT, supporting the option of a de-escalation therapy to treat HPV-positive OPSCC. The presence of few studies that reported oral intake outcomes, such as the time of the first oral intake after a TORS procedure, should be interesting to make new future studies.

In conclusion, few post-operative surgical complications were described in our analysis, with a mean of 5% complications per paper, without any statistical difference between tumor sites and tumor stage. Accordingly, Poupore et al. [54] did not evidence any differences in the recurrence rate and post-operative hemorrhages of the base tongue and tonsillar OPSCC in their patients treated with TORS. In our systematic review analysis, the most reported post-operative complication after the TORS procedure is dysphagia, which is mainly related to epiglottis removal [25]. To underline the huge importance of epiglottis integrity, as far as swallowing is concerned, we report that some specific surgical reconstructive techniques have been increasingly developed to aid the recovery of epiglottis function after its removal, such as sliding epiglottoplasty [55].

Therefore, future perspectives on the robotic management of OPSCC and laryngeal cancer should include the use of neoadjuvant CRT before TORS procedures. Sampieri et al. [56] described that the rule of neoadjuvant CRT followed by TORS for locoregionally advanced OPSCC achieved non-inferior survival outcomes compared to upfront surgery. However, these authors did not analyze the functional outcomes of this combined procedure. This systematic review exclusively analyzed SCC histology. However, it could be interesting to verify the same aims for other oropharyngeal histotypes, such as sarcomas, adenocarcinomas and mucoepidermoid tumors, through new papers [57]. At the same time, a comparison between functional outcomes of TORS to treat primary OPSCC and recurrent OPSCC can be performed in future studies. Furthermore, in order to better analyze the mentioned results, long-term validated and homogeneous parameters are needed. The lack of comprehensive guidelines for the management of OPSCC through TORS fosters inspiration for future studies.

## 5. Conclusions

TORS has evolved to a standard therapeutic modality for OPSCC, especially for T1 and T2 tumors and selected T3 ones. The robotic approach allows for similar oncological outcomes compared with surgical and nonsurgical techniques to treat OPSCC. Previous studies reported the superiority of TORS over traditional surgical approaches in terms of overall morbidity and swallowing outcomes. Furthermore, this study first demonstrated that TORS also presents favorable swallowing outcomes, better short- and long-term QoL outcomes and a lower prevalence and dependence on tracheostomy, FT and PEG than those reported in patients treated with CRT. However, the need for adjuvant therapies after TORS strongly reduced the advantage of robotic procedures, underlining how T1 and T2 tumors can mainly benefit from TORS alone.

## Figures and Tables

**Table 1:** Search string for each database.

Database	Search String	Articles Founds
Embase/Pubmed	(TORS OR “transoral robotic surgery”) AND (“Oropharyngeal Neoplasms”[Mesh]) AND (“Deglutition Disorders”[Mesh] OR “functional outcomes” OR “oral intake” OR speech OR “feeding tube” OR “naso gastric tube” OR “g tube” OR “percutaneous endoscopic gastrostomy” OR tracheotomy)	143
Scopus	(TORS) AND (functional outcome) AND (oropharyn*)	170
Web of Sciences	(TORS) AND (functional outcome) AND (oropharyn*)	232

**Table 2:** Tool for evaluating the methodological quality of case reports and case series. The eight questions included in the evaluation tool by Murad et al. [15] are categorized into the following four domains: selection, ascertainment, causality and reporting. Within each domain the Authors evaluated wheter the question related conditions were satisfied (1) or not (0). Final results differentiated case reports or series into 3 categories of risk of bias as follows: low risk (L) if all questions of the four domains were matched; intermediate risk (I) if questions of three out of the four domains were matched; high risk (H) if questions of less than three out of the four domains were matched.

Authors	Year	Selection	Ascertainment	Causality	Reporting	Risk of Bias
Achim et al. [16]	2017	1	1	1	1	L
Al Khudari et al. [17]	2013	0	1	0	0	H
Blanco et al. [18]	2013	0	1	0	0	H
Charters et al. [19]	2021	1	1	1	1	L
Dean et al. [20]	2010	1	1	1	0	I
Dziegielewski et al. [21]	2013	1	1	1	1	L
Genden et al. [22]	2011	1	1	1	0	I
Hughes et al. [23]	2023	1	1	1	1	L
Hurtuk et al. [24]	2011	0	1	0	0	H
Hutcheson etal. [25]	2019	1	1	1	1	L
Iseli et al. [26]	2009	0	1	0	0	H
Ji et al. [6]	2024	1	1	1	1	L
Lee et al. [27]	2014	1	1	1	0	I
Leonhardt et al. [28]	2010	1	1	1	1	L
Lorincz et al. [29]	2014	1	1	1	1	L
Li et al. [30]	2023	1	1	1	0	I
More et al. [31]	2015	0	1	1	0	H
Moore et al. [32]	2009	1	1	1	1	L
Moore et al. [33]	2012	1	1	1	1	L
Nichols et al. [34]	2019	1	1	1	1	L
Olsen et al. [35]	2013	1	1	1	1	L
Salmon et al. [36]	2021	1	1	1	1	L
Scott et al. [37]	2023	1	1	1	1	L
Sethia et al. [38]	2017	1	1	1	0	I
Sharma et al. [39]	2016	1	1	1	0	L
Shenouda et al. [40]	2019	1	1	1	0	I
Sievert et al. [41]	2020	1	1	1	0	I
Sinclair et al. [16]	2015	1	1	1	1	L
Van Abel et al. [10]	2019	1	1	1	1	L
Weinstein et al. [42]	2007	1	1	1	0	I
Weinstein et al. [9]	2010	1	1	1	0	I
Weinstein et al. [43]	2012	1	1	1	0	L
White et al. [44]	2013	1	1	1	1	L

## Data Availability

No new data were created or analyzed in this study.
